# Medical Education Manager: A Title Worthy of the Description

**DOI:** 10.7759/cureus.3373

**Published:** 2018-09-27

**Authors:** Kimberly R Gilfedder, Cara Giacomo, Julie Randall, Ginger L Wilson

**Affiliations:** 1 Medical Education and Simulation, University of Central Florida College of Medicine, Orlando, USA; 2 Internal Medicine, Greenwich Hospital - Yale New Haven Health System, Greenwich, USA; 3 Internal Medicine, Michigan State University College of Human Medicine, East Lansing, USA; 4 Internal Medicine, The University of Oklahoma School of Community Medicine, Oklahoma, USA

**Keywords:** gme, manager, administration, program, ume, coordinator, education, professional development, medical education manager

## Abstract

At a national meeting, an informal conversation took place between a group of medical education coordinators/administrators who reviewed and identified requisite skills needed for their vocation. Upon conclusion, it became evident that the profession was undervalued. It was also determined that retention and sustainability in this position is becoming transitory and that the need to raise awareness and construct a professional identity is crucial.

A nation-wide review of 30 institutions, literature searches, and national surveys revealed the need to construct a professional identity and tools for career growth. A focus group of medical education coordinators/administrators were tasked with the goal of creating a publication to encourage recognition and validity of this profession. The growth potential within the position needs to be elevated to a higher level with greater advantage for medical education coordinators/administrators. There are certifications available for both undergraduate medical education (UME) and graduate medical education (GME); however, results in achieving these qualifications are shown to be more of a personal satisfaction rather than enhancing career growth. Due to this insufficiency, medical education coordinators/administrators will look for other employment opportunities to advance their careers. In order to retain talented coordinators/administrators, there needs to be an element of growth opportunity in place for them to advance. Other careers provide growth opportunities to retain their valuable assets. Thus, it would appear logical that the same opportunities are made available to medical education coordinators/administrators.

The job responsibilities of program coordinators/administrators are determined to be diverse in nature. Therefore, it is apparent that the role is important to the success of any medical education program and identifies as a true “profession”.

Research shows the identity of the medical education coordinators/administrators is moving from “administrator-coordinator” towards academic managers, which more accurately depicts their role.

The administrative role of managing medical education programs has evolved tremendously with the need for a multilateral approach to develop a new job title and description. It is essential that institutions recognize administrators for the integral management positions they hold within a training program to help make it successful.

## Editorial

Medical education (ME) encompasses multidisciplinary clinical and non-clinical professionals who prepare, train, and support physicians as doctors of independent practice. Due to the nature of this ambiguous title, the vital functions of the administrative professionals often are undervalued; therefore, the purpose of this paper is to construct a professional identity and raise awareness of this important position. Medical education coordinators/administrators require an immense amount of training, as well as education. Medical education coordinators/administrators work as advisors and shepherds for burgeoning doctors and play an integral part in their professional development.

Upon entering a career in medicine, an individual will encounter a number of non-clinical and administrative processes. Thus, the hidden role of the medical education coordinator/administrator comes into play. This administrative career goes undetected by the general populace, since the training of our medical students and resident physicians primarily overshadows it. This non-clinical workforce consists of women and men who are the foundation of undergraduate medical education (UME)/graduate medical education (GME).

Background

An informal conversation between of a group of medical education coordinators/administrators during a national meeting reviewed and identified requisite skills needed for this position. The same informal group established that the dissemination of this information would raise awareness of the potential of medical education program administration as a credible and viable career path. Thus, the purpose of raising awareness is to enhance and support professional development by providing job description standards. Highly skilled medical education coordinators/administrators are essential to the success of programs and support the development of medical students, resident physicians, and fellows throughout the country.

The title of medical education manager enhances the position, rather than the current title of clerkship coordinator or program administrator. Historically, the title of clerkship coordinator/program administrator has been used in many disciplines, especially in health care and education. Thus, the title lends itself to vast interpretation of what UME/GME coordinators/administrators do. Furthermore, the argument to change the position title will accurately reflect the leadership and experience needed to fulfill the role. Essentially, a UME/GME administrator’s skill set includes, but is not limited to, exceptional written, verbal, and electronic communication skills, strong problem-solving/decision-making capabilities, and goal setting and supervisory skills. An administrator must possess high degrees of commitment and motivation, while seeking opportunities for additional responsibilities and promotion [1].

Experiences and responsibilities

In reviewing the Clerkship Directors in Internal Medicine (CDIM) Administrator Surveys (Engle K, Quirk KL, unpublished data 2013 and 2015), the data revealed vast discrepancies noted in the position’s job responsibilities. For example, we identified that each job description has more than 230 different responsibilities documented. When identifying broad classifications, it proved to be challenging, considering many responsibilities were similar but designated by different descriptions of the tasks.

A successful coordinator/administrator requires a unique skill set. High-performing characteristics in this position are the ability to work with diverse populations of traditional and non-traditional students/residents, supervise the complex daily operations of the medical school and residency training programs while simultaneously meeting critical deadlines. Additionally, coordinators/administrators interface with team leaders and institutional administration to comply with program policies, procedures, and accreditation needs. Thus, a candidate’s level of work experience and educational background needs to be carefully and thoroughly vetted in order to meet the criteria for the position.

Accumulated diversity 

Surveys reveal that employers across the country have educational diversity when hiring for this position. At some institutions, a high school diploma is all that is necessary, while other institutions require a minimum of an undergraduate degree to a doctorate degree. National certifications, such as Training Administrators of Graduate Medical Education (TAGME) or Administrators Certification in Undergraduate Medical Education (ACUME), are available to further enhance depth of knowledge, skills, and education. Therefore, these certifications provide professional recognition and credibility at the institutional, state, and national levels.

In addition to educational imparity, there are inconsistencies in job titles for this position. Once the variation was identified, a task force was formed in 2015 by medical education coordinators/administrators to analyze and construct a position paper to develop a defined path to disseminate information to the general public. Based on our research, the task force suggests adopting the title of medical education manager by all institutions.

Careers and crossover

**Figure 1:**
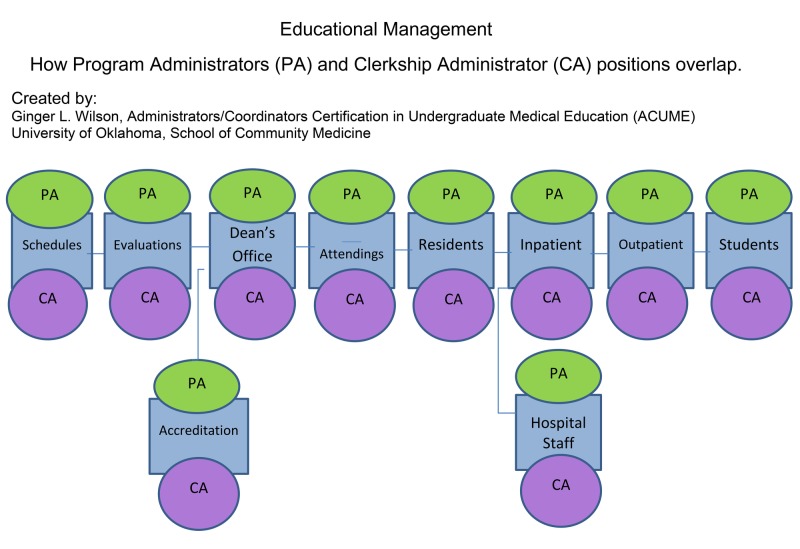
Educational Management

UME and GME administrator roles overlap in terms of skill set similarities that enable them to provide a structured, educational environment for the benefit of medical students and residents; please see Figure 1 below [2] [3].

Together, they function as liaisons among clerkship and program directors, attending physicians, residents, fellows and medical students, as well as institutional administrative departments that interface with the medical school and sponsoring institution (e.g., risk management, institutional review board). Student and resident programs run parallel to each other, since each has an accreditation body that ensures standards are being met across the country. What is not obvious to most is the understanding of what makes the two programs distinguishable and how much they intersect with one another. For example, administrative changes made to GME programs have an immediate effect on UME programs. Therefore, a reduction in the number of hospital teams and/or changes to the number of attending physicians can have a direct impact on both programs.

Under the heading “Other Duties as Needed,” this category further identifies the role of the program administrator/coordinator as trainee advocate, listener, career counselor and coach, indicative of some of the other responsibilities a manager takes on. Therefore, professional development and educational training are important in order to be effective in this role. It is essential that students and residency coordinators/administrators work together and network, since the primary goal of the latter is to prepare medical students for postgraduate training in GME programs.

Career satisfaction

The Association of Program Directors in Internal Medicine (APDIM) also produced administrator surveys (Sheehan S, unpublished data, 2015 and Speilmann K, unpublished data, 2017) that outlined the pros and cons of the profession, including job satisfaction and recognition as well as a varied set of questions including residency size, job experience, job titles, GME system, region, and education level. The surveys revealed that regional demographics (e.g., metropolitan versus suburban locations) did not allow for cost-of-living variances. Additionally, the surveys did ask specific questions that produced results about membership demographics, title, tenure, education level, program size, institution type, and salary. However, the surveys did not address whether or not national certification served as a factor in recruitment for the position and the effect of tenure on salary and title. The results were compared regionally, along with one area outside the United States. What was remarkably different between the CDIM and APDIM surveys was that APDIM asked questions regarding professional burnout, organized teaching, and professional development. In our opinion, the data relating to professional burnout and a desire to resign was most revealing; as a consequence, lack of professional support by the sponsoring institution can create significant hiring gaps in order to maintain continuity in the career.

Recommendations

The purpose of this publication is to heighten awareness regarding the role of the medical education administrator/coordinator. The profession can no longer sustain itself in the current academic climate without change. It is of vital importance to pursue a nationally recognized job title, job description, and salary structure in order to promote sustainability in the industry. First, we recommend assigning levels to the given titles, dependent upon years of experience, program and resident complement, and compensation commensurate with experience and/or education. Standardizing reporting and promotion structure will lead to less employment attrition, strengthen position training, and create promotional incentives. An example of standardization is outlined in Table 1 below.

**Table 1:** Medical Education Program Administrator/Manager Career Ladder Model UME - Undergraduate medical education. GME - Graduate medical education.

Medical Education Program Administrator/Manager				
Career Ladder Model				
(Salaried Positions)				
Title	Education	Years Experience	Certification	Skills
Program Administrator/Manager I	Associate of Arts (AA) Degree or years of experience, preferred	Full time employee, Preference of 1-2 years of program manager experience	No certification required	*Focus on verbal skills, written skills, and knowledge development
				*Consults with more experienced team members when necessary
				*Exhibits basic knowledge of Accreditation Council for Graduate Medical Education (ACGME) common program requirements
				*Exhibits basic knowledge of ACGME specialty requirements
				*Exhibits basic knowledge of professional ethics, laws, and regulations
				*Demonstrates entry level competency in administrative management of program
				*Demonstrates entry level competency in fiscal management of program
				*Demonstrates entry level competency in data management of program
				*Demonstrates entry level competency in program compliance management
Program Administrator/Manager II	AA Degree or years of experience, preferred	Full time employee, Preference of 3-5 years of program manager experience	No certification required	*Demonstrates characteristics of Program Administrator/Manager I plus:
				*Works efficiently under the direction of experienced team leader
				*Participates in Departmental Medical Education Committee meetings
				*Focuses on obtaining additional knowledge and skills
				*Exhibits working knowledge of ACGME common program requirements
				*Exhibit working knowledge of ACGME specialty requirements
				*Exhibits working knowledge of professional ethics, laws and regulations
				*Demonstrates competency in administrative management of program
				*Demonstrates competency in fiscal management of program
				*Demonstrates competency in data management of program
				*Demonstrates competency in program compliance management
				*Member of professional organization, no certification required
Program Administrator/Manager III	Bachelor of Arts (BA) or Bachelor of Science (BS) Degree or years of experience, preferred	Full time employee, Preference of 5 years of program manager experience	Studying for certification	*Demonstrates characteristics of Program Administrator/Manager II plus:
				*Works independently, self-motivated
				*Demonstrates ability to multitask in coordinating multiple, simultaneous program demands
				*Exhibits excellent knowledge of ACGME common program requirements
				*Exhibits excellent knowledge of ACGME specialty requirements
				*Exhibits excellent knowledge of professional ethics, laws and regulations
				*Demonstrates expertise in administrative management of program
				*Demonstrates expertise in fiscal management of program
				*Demonstrates expertise in data management of program
				*Demonstrates expertise in program compliance management
				*Ability to run program faculty education meetings with continuous follow-up and action planning
				*Ability to monitor remediation compliance
				*Attendance in at least one national meeting in the past 2 years
				*Active committee membership in professional organization
				*Participation in quality systems assessment and offers suggestions for improvement in program operations
				*Development and presentation of institutional programs such as in-service training sessions
				*Participation in institutional mentorship program
Program Administrator/Manager IV	BA or BS Degree or years of experience, preferred	Full time employee, preference greater than of 5 years of program manager experience	Global Certification	*Demonstrates characteristics of Program Administrator/Manager III plus:
				*Committee leadership in professional organization.
				*Participates in educational research
				*Participates in institutional initiatives
				*Development and presentation of departmental programs
				*Program specific certification
				*Leader in institutional Mentorship Program
				*Participation in national mentorship program
				*Invited speaker - national level conference
Administrative Director for Medical Education (Undergraduate Medical Education [UME] and Graduate Medical Education [GME])	BA or BS Degree or years of experience, preferred	Full time employee, preference of 7 years of program manager experience	Certification in Institutional Professional & Global	*Responsible for program and institutional regulatory compliance for all GME and UME programs
				*Fiscal management and responsibility for institutional, GME and UME budgets
				*Expertise in institutional, ACGME Common, GME program specific and UME requirements
				*Expertise in program review, action plan creation and oversight to meet regulatory compliance
				*Leadership role in mentoring program, problem solving and conflict resolution
				*Administrative management of GME and UME Program Administrators including oversight, professional development, review, promotion, hiring and with approval and sign off level and Medicare Cost reporting management
				*Program Data Management oversight and review for institutional, GME and UME programs in meeting regulatory compliance
				*Developing and implementing programs to enhance GME, UME and institutional compliance
				*Leader in Orientation and Mentorship program for new administrators and program directors
				*National leader in the development of programs, educational research and publications
				*National Conference Invited faculty and/or panel expert
				*National Board Chair position

Second, the authors agree that long-term recommendations need to be developed, implemented, and sustained by creating a national task force with representation from both undergraduate and graduate medical education. This national task force will be charged with the development of a strategic plan to strengthen and enhance current initiatives indicated in this paper. Furthermore, the authors recommend presenting the findings to stakeholders outside of academic medicine (e.g., Society for Human Resource Management).

Third, once these findings are established, our recommendation is to further develop a curriculum and collaborate with educational institutions to offer a post-baccalaureate certification program. Instructor training for experienced coordinators/administrators will be offered to teach continuing medical education manager courses to further encourage and enhance ACUME/TAGME certification and maintain sustainability in the profession.

Conclusion

As demonstrated in this paper and our daily commitment in support of our developing physicians, the disparity demonstrated in our various job titles does not accurately reflect the service we provide to our programs and institutions. Therefore, it is necessary to readdress the continuously changing roles and responsibilities by pursing a nationally recognized job title and commensurate salary structure.
